# Lasalle County Medical Society

**Published:** 1853-08

**Authors:** C. Hard, J. O. Harris

**Affiliations:** President; Secretary


*Lasalle County Medical Society.*

Pursuant to a notice given through the public prints, and otherwise, a meeting of physicians was held at Ottawa, on the 29th ult.

On motion, Dr. C. Hard was appointed President of the meeting, and Dr. J. O. Harris Secretary.

After the object of the meeting had been stated by the President, the following persons were appointed a Committee to prepare a set of By-Laws for the governance of the proposed Society, viz : Drs. J. O. Harris, P. Kirwin, J. Hay, R, McArthur, and A. Hard.

On motion the meeting adjourned to meet again at 3 P. M. Meeting called to order by the President.

Dr. Harris, the chairman of the Committee on By-Laws, reported that they had performed the duty assigned them, which report was accepted, and the By-Laws presented were, with some amendments, adopted.

The following officers were then elected for the current year, viz : Dr. C. Hard, President ; Dr. E. S. Morey, Vice President ; Dr. J. O. Harris, Secretary and Librarian ; Dr. P. Kirwin, Treasurer, and Drs. T. Hay, P. Kirwin, C. Haud, E. S. Morey, and J. O. Harris, Censors.

On motion, the Secretary was requested to publish in such journals as he thought proper, an abstract of the proceedings of this meeting, when

On motion the society adjourned to meet at Ottawa on Wednesday the 7th of September next.

C. HARD,
*President.*

J. O. HARRIS,
*Secretary.*

It may be proper to add that there has been for a year or more a *City* Medical Society in existence at Lasalle, but until the present time no County Society was ever organized. The meeting was not at all attended as was anticipated, yet, from the interest manifested by these who were present and from assurances given by others who were unavoidably prevented from attending, we feel confident that the society will flourish and be productive of much good. J. O. HARRIS.

